# Experiences with the European guidelines on quality criteria for radiographic images in Tanzania

**DOI:** 10.1120/jacmp.v2i4.2601

**Published:** 2001-09-01

**Authors:** W. E. Muhogora, A. M. Nyanda, R. R. Kazema

**Affiliations:** ^1^ Department of Radiation Protection Services National Radiation Commission P.O. Box 743 Arusha Tanzania; ^2^ Department of Radiology and Medical Imaging Muhimbili National Hospital P.O. Box 65000 Dar es Salaam Tanzania

**Keywords:** diagnostic radiology, image quality, European guidelines

## Abstract

Objective assessment of the quality of radiographic images is practically a difficult task and protocols that address this problem are few. In 1996, the European union published nearly objective image quality criteria to unify the practices in Europe. However, experience with these criteria in countries of lower health care levels is little documented. As a case study in Tanzania, we present the general performance of European guidelines in some Tanzanian hospitals to a total of 200 radiographs obtained from some common *x*‐ray examinations. The results show that more than 70% of chest (PA), lumbar spine (AP), and pelvis AP radiographs passed the quality criteria, while the performance of lumbar spine LAT *x*‐ray examinations was about 50% and therefore less satisfactory. The corresponding mean entrance dose to the patient for specified *x*‐ray techniques was of range 0.08–0.56 mGy, 3.1–7.7 mGy, 2.53–5.4 mGy, and 4.0–16.78 mGy for chest PA, lumbar spine AP, pelvis AP and lumbar spine LAT *x*‐ray examinations, respectively. Although a good number of observers were not well familiar to the guidelines, the quality criteria have been found useful and their adoption in the country recommended. The need to provide relevant education and training to staff in the radiology departments is of utmost importance.

PACS number(s): 87.57.–s, 87.52.–g

## INTRODUCTION

Despite a small but increasing hazard of diagnostic *x*‐rays to human beings,^1,^, ^2^ studies aimed at achieving low patient doses with sufficient image quality have continued to be the area of research interest. The relationship between the quality of radiographic image and dose to patient is known to depend on the performance characteristics of the *x*‐ray equipment, patient shape and size, type of image receptors, radiographic techniques, viewing conditions as well as staff experiences.^3,^, ^4^ Protocols that objectively establish this relationship, are however, few worldwide and inexistent in many countries such as Tanzania. Recently, the European union has published a set of nearly objective guidelines for good radiographic techniques and the corresponding level of the image quality.^5^ The guidelines have proved to be a useful tool to unify the practices in Europe. In attempts to address the problem on dose reduction without affecting the patient care, a research on radiation protection in diagnostic radiology was initiated in Tanzania. The hospitals under study were MMC (Muhimbili Medical Center), KCMC (Kilimanjaro Christian Medical Center), BMC (Bugando Medical Center), MCH (Mbeya Consultant Hospital), and ARH (Arusha Regional Hospital). A study on film rejects rate analysis revealed varying radiographic techniques and skills (positioning, centering, tube voltage, and tube current‐time product selection), as well as patient influences as mainly responsible to more than 90% of waste film.^6^ The average magnitude of waste film per week for a given *x*‐ray examination was as summarized in Table I.

**Table I:** The average number of waste film per week for a given examination.

Hospital	Number of *x*‐ray rooms	Number of waste film per week			
		Chest AP	Lumbar spine AP	Lumbar spine LAT	Pelvis AP
MMC	5	54	8	25	63
KCMC	3	23	5	2	3
BMC	3	13	3	8	9
MCH	1	20	3	5	14
ARH	1	3	3	3	2

Patient dose assessment identified four methods that could reduce unnecessary dose to patients. The measures included lowering tube current‐time product, increasing tube voltage, increasing filtration, increasing the image receptor speed, and improving the processing conditions.^7^ During this study, up to 50% of radiographs were rated poor based on European guidelines on quality criteria for radiographic images. Following this magnitude of poor radiographs, consultancy with radiologists in attendance of radiographers was made on possible strategies to reduce the waste film level. Chief amongst the strategies were the use of 400‐speed class of film‐screen combination instead of the usual 200‐speed class, improvement and maintenance of film processing conditions, use of exposure charts, and adherence to the recommended practices of good imaging. Later on, the lowering of tube current‐time product and increasing the speed of film‐screen combination and optimizing film processing conditions were practically demonstrated to be the most effective dose reduction measures in the country.^8^ Recently, a follow‐up study on the effectiveness of dose reduction measures in general clinical conditions as a reliable indicator for optimization has been reported, where patient doses were reduced up to 77%.^9^ During this follow‐up study, the quality of radiographic images obtained after the implementation of dose reduction measures was again evaluated using the European quality criteria and the results are hereby presented. The fact that no similar study has been done in the country, the foreseen research results could provide first experience on the subject in the country and additional experience elsewhere.

## METHODS

The study was conducted at the same hospitals using the same *x*‐ray equipment and film processors as during previous studies.^6,^, ^7,^, ^8,^, ^9^ Four *x*‐ray projections namely chest (PA), pelvis (AP), lumbar spine (AP), and lateral (LAT) were employed for the intended study. Ten adult patients of weight ranging from 60 to 80 kg and thickness between 17 and 25 cm were selected for each of the four *x*‐ray projections at each hospital giving a collective sample of 200 patients. The employed *x*‐ray equipment had their tube voltage and tube current‐time product settings verified by noninvasive *x*‐ray test device, Victoreen model 4000M+. The film processing conditions at each hospital were also optimized and maintained accordingly.^8^ This implies that the study was conducted to the *x*‐ray equipment whose performance was sufficiently known. Details on *x*‐ray equipment employed are summarized in Table II. During *x*‐ray examination, the requirements and technical parameters of good imaging were closely followed as recommended.^4^ The mean ESD ranges and the corresponding summary of selected techniques at each hospital are presented in Table III. The quality of the obtained radiographs per *x*‐ray projection was tested for compliance with the European guidelines on quality criteria for radiographic images^5^ by five well‐experienced radiologists. Three observers (including one of the authors, R.R.K.) are consultant radiologists with academic qualification at a postgraduate level in radiology and a working experience of over ten years. The other two observers have postgraduate diploma in radiology and have worked for three years under a consultant radiologist and over four years independently. According to the European guidelines, the image criteria refer to characteristic features of imagined anatomic structures of each radiograph with a specific degree of visibility. The observers evaluated the image quality of all ten radiographs of each *x*‐ray projection (according to the basis indicated in Table IV) against all anatomical structures (Tables V–VIII). Along with image quality assessment using the European quality criteria, optical densities of a sample of radiographs were also measured using a RMI densitometer, serial number 211–2176F to establish typical values for achieved optimized conditions.

**Table II:** Type of x‐ray equipment and film processors at hospitals.

Hospital	X‐ray machine	Processor	Focus to film distance (FFD)	HVL at 80 kVp (mm Al)
MMC	Phillips, model Emerald 125	automatic, Kodak RPX Omat model M6B	150 cm: chest PA 100 cm: rest *x*‐ray examinations	3.1
	Phillips, model U‐5BC‐41T			3.4
KCMC	Phillips, model Emerald 125 RPX Omat	automatic, Kodak model M6B	150 cm: chest PA 100 cm: rest *x*‐ray examinations	4.0^a^
	Phillips, model Emerald 125			4.7
BMC	Siemens, model 125/20/40–80	manual	150 cm: chest PA 100 cm: rest *x*‐ray examinations	2.8
	Shimadzu, model Circlex 1/2P/18C			3.7
MCH	Shimadzu, model Circlex 1/2P/80–806	manual	150 cm: chest PA 100 cm: rest *x*‐ray examinations	2.1
ARH	Phillips medio 51, model Rotalix	manual	150 cm: chest PA 100 cm: rest *x*‐ray examinations	3.6

a HVL measurements done at 120 kVp.

**Table III:** Mean entrance surface dose to patients at hospitals.

Hospital	*x*‐ray examination	Mean tube voltage range (kVp)	Mean tube current‐time product range (mAs)	Speed class of film‐screen combination	Mean ESD range (mGy)
MMC	chest PA	76–83	9.8–22	400/200	0.2–0.36
	pelvis AP	75–80	120–143.7	400/200	2.53–5.4
	lumbar spine AP	70–80	89–120	400/200	3.87–4.94
	lumbar spine LATERAL	79–80	207.1–240.7	400/200	10.26–16.78
KCMC	chest PA pelvis AP	120	1.2	400	0.08–0.01a
	lumbar spine AP lumbar spine LATERAL	70–73	55	400	3.84–4.60b
BMC	chest PA	59.7–68.5	25.3–40	400/200	0.22–0.39
	pelvis AP	77–81	125	400/200	2.9–3.1
	lumbar spine AP	88–90	151.7–180	200	6.5–7.7
	lumbar spine LATERAL	90–120.4	160–250	400	9.83–14.4
ARH	chest PA	70–87.5	10–21.6	200	0.20–0.34c
	pelvis AP	90	33–35	200	3.0–3.9c
	lumbar spine AP	80–90	70–80	400/200	3.1–3.3c
	lumbar spine LATERAL	90	70–90	400/200	4.0–6.2

Note: Some ESD ranges include patient doses obtained with additional filtration of 1 mm Al, hence HVL of $4.7^{a}$, $4.9^{b}$, and $3.8^{c}\text{mm}$ Al (see also Table II).

**Table IV:** The basis employed during the assessment of the quality of radiographic images.

Image criteria	Degree of visibility	Score
‐Visualization of characteristic features.	‐feature detected and fully reproduced	‐good
	‐feature just visible	‐satisfactory
	‐feature not visible	‐poor
‐Reproduction of anatomical structures.	‐detail visible and clearly defined	‐good
	‐detail not visible or not clear	‐poor
‐Visually sharp reproduction	‐details clearly defined	‐good
	‐details just clear	‐satisfactory
	‐details not clear	‐poor

## RESULTS AND DISCUSSIONS

Tables V to VIII give the results of image quality assessment based on the European guidelines. Typical densitometry results on 400‐speed class of film screen combination at each hospital are given in Table IX. From Tables V to VIII, it can be seen that the scores rated good (i.e., all anatomical structures seen) were in percentage 39%, 31.2%, 25%, and 2.7% for chest PA, pelvis AP, lumbar spine AP, and lumbar spine LAT, respectively. On the other hand, the number of poor scores led in lumbar spine LAT projections (53%) followed by pelvis AP *x*‐ray examinations (28%). For lumbar spine AP and chest PA *x*‐ray examinations, the poor scores were 21% and 20%, respectively. Despite poor performance in lumbar spine LAT and pelvis AP *x*‐ray projections, such situation did not necessarily constitute to waste film. The majority of radiographs rated satisfactory could still meet the clinical needs of the intended diagnosis. The low optimization levels of film processing conditions, different levels of experiences among the radiology staff, and variable *x*‐ray techniques form a possible explanation for such performance in lumbar spine LAT and pelvis AP projections. The data suggest that the image quality criteria for good pelvis AP and lumbar spine LAT *x*‐ray examinations were the most difficult to meet, although this observation is not reflected in the list of image criteria that could be seen with some difficulties (Table X). The difficulty is attributable to varying technical and clinical needs. The majority of observers noted the detailed nature of the quality criteria and were of opinion that the variations in *x*‐ray techniques and film processing conditions were the main source of significant numbers of radiographs rated satisfactory that otherwise could be good radiographs. The observers also noted the difficulty of seeing image criteria as a function of increasing film contrast requirements.

**Table V:** Compliance with European guidelines for 50 chest PA x‐ray examinations.

Image criteria	Number of scores per score category^a^		
	*Good*	*Satisfactory*	*Poor*
‐Performed at deep inspiration (as assessed by the position of the ribs above the diaphragm either 6 anteriorly or 10 posteriorly)	50	0	0
‐Symmetrical reproduction of the thorax	14	30	6
‐Medial border of the scapulae to be outside the lung field	0	25	25
‐Reproduction of the whole rib cage above the diaphragm	21	19	5
‐Reproduction of the vascular pattern in the whole lung particularly the peripheral vessels	12	28	10
‐Visually sharp reproduction of			
(a) the trachea and proximal bronchi, the borders of the heart and aorta	15	10	12
(b) the diaphragm and costo‐phranic angles	13	12	8
‐Visualization of the retrocardia lung and the mediastinum	17	26	6

a ‐good: feature detected and fully reproduced, detail visible and clearly defined ‐satisfactory: feature just visible, detail just visible but not clearly defined ‐poor: feature invisible, detail invisible or not clear

**Table VI:** Compliance with European guidelines for 50 lumbar spine AP x‐ray examinations.

Image criteria	Number of scores per score category^a^		
	*Good*	*Satisfactory*	*Poor*
‐Linear reproduction of the upper and lower plate surfaces in the centered beam area and visualization of the intervertebral spaces	17	33	0
‐Visually sharp reproduction of the pedicles	0	34	16
‐Visualization of the intervertebral joints	26	24	0
‐Reproduction of the spinuos and transverse process	23	10	17
‐Visually sharp reproduction of the cortex and trabecular structures	0	40	10
‐Reproduction of the adjacent soft tissues, particularly the psoas shadows	8	22	20

a ‐good: feature detected and fully reproduced, detail visible and clearly defined ‐satisfactory: feature just visible, detail just visible but not clearly defined ‐poor: feature invisible, detail invisible or not clear

**Table VII:** Compliance with European guidelines for 50 lumbar spine LAT x‐ray examinations.

Image criteria	Number of scores per score category^a^		
	*Good*	*Satisfactory*	*Poor*
‐Reproduction by tangential projection of the inferior end plate of L5 and the superior end of S1	4	20	26
‐Visualization of the anterior border of the upper scrum	0	36	14
‐Reproduction of vertebral pieces of the upper scrum	0	10	40

a ‐good: feature detected and fully reproduced, detail visible and clearly defined ‐satisfactory: feature just visible, detail just visible but not clearly defined ‐poor: feature invisible, detail invisible or not clear

**Table VIII:** Compliance with European guidelines for 50 pelvis AP x‐ray examinations.

Image criteria	Number of scores per score category^a^		
	*Good*	*Satisfactory*	*Poor*
‐Symmetrical reproductionofthe pelvis	30	20	0
‐Visualization of the sacrum and its intervertebral foramina	10	14	26
‐Visualization of the sacroiliac joints	12	20	18
‐Reproduction of the neck of the femora which should not be distorted by foreshortening or rotation	0	40	10
‐Reproduction of spongiosa and corticalis and visualization of the trochanters	26	8	16

a ‐good: feature detected and fully reproduced, detail visible and clearly defined ‐satisfactory: feature just visible, detail just visible but not clearly defined ‐poor: feature invisible, detail invisible or not clear

**Table IX:** Typical film optical density ranges of optimized radiographs on 400‐speed class of film‐screen combination.

X‐ray examination	Hospital	Film density range	Mean film density^a^
Chest PA	MMC	0.35–2.14	1.1±0.41
Chest PA	KCMC	0.25–1.40	0.7±0.2
Chest PA	BMC	0.51–1.80	1.62±0.16
Chest PA	MCH	0.28–1.32	0.76±0.35
Pelvis AP	MMC	1.67–2.33	1.9±0.25
Pelvis AP	KCMC	1.0–2.4	1.4±0.3
Pelvis AP	BMC	0.22–2.36	1.21±0.56
Pelvis AP	MCH	0.46–1.86	1.15±0.60
Lumbar spine AP	MMC	1.02–1.75	1.35±0.28
Lumbar spine AP	KCMC	0.8–1.6	1.2±0.1
Lumbar spine AP	BMC	0.47–1.78	0.98±0.43
Lumbar spine AP	MCH	0.40–1.24	0.82±0.29
Lumbar spine LAT	MMC	1.25–2.33	1.81±0.53
Lumbar spine LAT	KCMC	0.7–2.5	1.2±0.4
Lumbar spine LAT	MCH	0.46–1.98	1.06±0.5

a At one standard deviation.

**Table X:** Specific image criteria, which were generally difficult to see.

X‐ray examination	Image criteria	Reference hospital(s)	Number of radiographs
Chest PA	‐symmetrical reproduction of the thorax	‐KCMC	4
	‐reproduction of the whole rib cage above the diaphragm	‐KCMC, MCH, ARH	8
	‐Visualization of the retrocardiac lung and the mediastinum	‐MMC, KCMC	6
	‐Visually sharp reproduction of		
	(a) the trachea and proximal bronchi, the borders of the heart and aorta	‐MMC, ARH	3
	(b) the diaphragm and casto‐phrenic angles	‐MCH, ARH	8
Lumbar spine AP	‐Visually sharp reproduction of the Pedicles	‐MMC, KCMC	5
	‐Reproduction of the spinious and and transverse processes	‐MMC, KCMC	8
	Reproduction of the adjacent soft tissues particularly the psoas shadows	‐KCMC, ARH	6
Lumbar spine LAT	‐Reproduction by tangential projection of the inferior end plate of L5 and the superior end plate of S1	‐MMC	3
	‐Visualization of the anterior border of the upper sacrum	‐MMC	5
	‐Reproduction of vertebral pieces of the upper sacrum	‐MMC	5
Pelvis AP	‐Symmetrical reproduction of the pelvis	‐MMC, KCMC	6
	‐visualization of the sacrum and its intervertebral foramina	‐MMC, KCMC, ARH	10
	‐Visualization of the sacroiliac joints	‐ARH	3
	‐Reproduction of spongiosa and and corticalis and visualization of the trochanters	‐MCH	4

The two observations above suggest that the application of the quality criteria can be prohibitive in lower healthcare levels (e.g., district hospitals, health centers, dispensaries, etc.) where radiographs reading and optimized film processing conditions are normally less satisfactory. However, some of these “difficult criteria” were not a prerequisite under the type of projections requested. For example, “visualization of the retro cardiac lung and the mediastinum” could better be seen in lateral view of chest than in PA view (reference ARH). Likewise a “visual sharp reproduction of the pedicles of the pelvis, particularly the psoas shadows” needs a special view of lumbar spine LAT for better visualization than the ordinary lateral view, which was of interest (reference KCMC). Such two different experiences show the need to adopt the European guidelines after careful examination of different specific clinical situations.

The opinion has also been expressed by the publisher of the European guidelines, who clearly stated that the quality criteria define a level of performance considered necessary to produce images of standard quality for a particular anatomical region, without regard to specified clinical indication.^5^ Despite that, the overall results show that the majority of chest PA, lumbar spine AP, and pelvis AP radiographs passed the quality criteria by more than 70%, while the performance of lumbar spine LAT *x*‐ray examinations was slightly less than 50% and therefore less satisfactory. Generally, the compliance to European guidelines compare well with a similar study done in some African countries.^10^ The film densitometry results achieved in this study (Table IX) are also similar to the suggested density range values elsewhere.^3^ Excluding the data on lumbar spine LAT *x*‐ray examinations, the results (Tables V, VI, and VIII) indicate improvement on the performance of the European criteria from up to 50% of the radiographs rated poor earlier^7^ to 28% of poor radiographs observed during this study. The performance can implicitly be associated to the reduction of the number of retake films; the observation was later confirmed by the radiology staff.

## CONCLUSION

The performance of European guidelines on quality criteria for radiographic images in Tanzania has been presented. The usefulness of the criteria in optimization studies of *x*‐ray examinations has been demonstrated. The compliance of majority radiographs (obtained after implementation of dose reduction measures) to the guidelines is evidence that the radiation protection of patients was sufficiently optimized. The minimization of patient doses implies that the radiation risk associated with the *x*‐ray examinations was also minimized following the reduction in the number of waste film and hence retake films. Provided that the radiology staffs are familiar with the criteria and local clinical requirements are met, the European guidelines on quality criteria for radiographic images can practically be adopted in Tanzania. It is recommended that this study be extended to wider scale for reliable contribution to the medical regulations of ionizing radiation in pipeline. However, it is evident that relevant education and training programs must be initiated to the radiology staff for best results.

## ACKNOWLEDGMENTS

The work was supported by a grant from the International Atomic Energy (Contract No. 9336/RO). The authors are further grateful to the International Center for Theoretical Physics for providing the opportunity to one of them (W.M.) to undertake a scientific study visit related to the work in Trieste, Italy. Last, but not least, the authors are especially thankful to Dr. H. Dieffenthal (KCMC), Dr. J. J Lyimo (Arusha *x*‐ray center), Dr. L. Iseme, and Dr. Z. Nhanga (MCH) for evaluating the quality of radiographic images and valuable discussions.
